# Facts, values, and Attention-Deficit Hyperactivity Disorder (ADHD): an update on the controversies

**DOI:** 10.1186/1753-2000-3-1

**Published:** 2009-01-19

**Authors:** Erik Parens, Josephine Johnston

**Affiliations:** 1 The Hastings Center, 21 Malcolm Gordon Road, Garrison, New York 10524, USA

## Abstract

The Hastings Center, a bioethics research institute, is holding a series of 5 workshops to examine the controversies surrounding the use of medication to treat emotional and behavioral disturbances in children. These workshops bring together clinicians, researchers, scholars, and advocates with diverse perspectives and from diverse fields. Our first commentary in CAPMH, which grew out of our first workshop, explained our method and explored the controversies in general. This commentary, which grows out of our second workshop, explains why informed people can disagree about ADHD diagnosis and treatment. Based on what workshop participants said and our understanding of the literature, we make 8 points. (1) The ADHD label is based on the interpretation of a heterogeneous set of symptoms that cause impairment. (2) Because symptoms and impairments are dimensional, there is an inevitable "zone of ambiguity," which reasonable people will interpret differently. (3) Many other variables, from different systems and tools of diagnosis to different parenting styles and expectations, also help explain why behaviors associated with ADHD can be interpreted differently. (4) Because people hold competing views about the proper goals of psychiatry and parenting, some people will be more, and others less, concerned about treating children in the zone of ambiguity. (5) To recognize that nature has written no bright line between impaired and unimpaired children, and that it is the responsibility of humans to choose who should receive a diagnosis, does not diminish the significance of ADHD. (6) Once ADHD is diagnosed, the facts surrounding the most effective treatment are complicated and incomplete; contrary to some popular wisdom, behavioral treatments, alone or in combination with low doses of medication, can be effective in the long-term reduction of core ADHD symptoms *and *at improving many aspects of overall functioning. (7) Especially when a child occupies the zone of ambiguity, different people will emphasize different values embedded in the pharmacological and behavioral approaches. (8) Truly informed decision-making requires that parents (and to the extent they are able, children) have some sense of the complicated and incomplete facts regarding the diagnosis and treatment of ADHD.

## Background

The US Centers for Disease Control estimates that approximately 4.6 million (8.4%) American children aged 6–17 years have at some point in their lives received a diagnosis of Attention-Deficit/Hyperactivity Disorder (ADHD). Of these children, nearly 59% are reported to be taking a prescription medication [1]. Rates of stimulant use have been growing fast in both the US and Europe [2-4]. Indeed, in the last 10 years, Germany has seen a 47-fold increase [5]. But per capita stimulant consumption remains greater in the US than in all of Europe. According to the International Narcotics Control board [6], "The per capita consumption of methylphenidate in the US between 2003 and 2005 was approximately six times greater than that of Australia, eight times greater than that of Spain, and 18 times greater than that of Chile" [7].

Not just school-age children are being treated with stimulants. Stimulant use among preschool children is also greater in the US than elsewhere: 0.44% of preschoolers in the US are prescribed stimulants, compared with 0.05% of preschoolers in the Netherlands, 0.02% of preschoolers in Germany, and 0% of preschoolers in the UK [8].

The duration of treatment and complexity of the treatment regimen is also growing. Before 2000, most children treated for ADHD received short-acting drugs, during school, for 1 or 2 years. Today many receive long-acting drugs while in – and out – of school and the prevailing recommendation from ADHD experts is to start medication early and to continue as long as medication is needed. This suggests that, if they adhere to their regimens, many American children diagnosed with ADHD will receive far higher lifetime doses than similar children in the past [9]. Even outside the US, a study of Dutch youths showed that between 1995 and 1999, duration of exposure to stimulants increased [10]. In addition, children are more likely than in the past to have more than one diagnosis and therefore to be taking multiple medications simultaneously [11].

Even without any further increase in the rate of stimulant use (data from a federal survey suggest it may be leveling off [12], whereas Health Management Organization population-based data show a slight but continuing increase [4]) current usage rates raise a range of questions concerning how we conceive of what we call ADHD in the US and what are the most effective and appropriate ways to respond to children who receive that diagnosis. Some of these questions can be answered by more research and better facts. Other questions turn on values. Some are peculiar to the diagnosis and treatment of ADHD, but most are questions that also arise in the diagnosis and treatment of other behavioral and emotional disturbances for which ADHD is a valuable case study [13].

## The ADHD label refers to a heterogeneous set of phenomena

Some manifestations of the behaviors that today we call symptoms of ADHD (inattention, hyperactivity, and impulsivity) have been recognized as problematic for the last 100 years – and, arguably, for much longer. Generally, children are brought to their physicians because parents or teachers are concerned that the child's behavior is preventing him or her from functioning normally at home, in school, or in other settings. In the majority of cases, teachers are the first to suggest that a child might have ADHD [14]. Initial assessments are often carried out by school psychologists or clinical psychologists before a referral is made to a physician. Workshop participant and educational psychologist Roy Martin noted: "In the vast majority of cases, that physician is a pediatrician. In my experience only 5 to 10% of cases result in a specialized referral to a psychiatrist." Because physicians do not observe the child's behavior in school or at home, they must rely heavily on parents' and teachers' reports. According to Martin, "Physicians are under pressure to try to help, and therefore tend to respond to the felt needs of parents and teachers." That response often takes the form of a diagnosis, which physicians base on their training, clinical judgment, and experience, as well as on diagnostic tools and guidelines, such as those in the American Psychiatric Association's Diagnostic and Statistical Manual (DSM).

According to the fourth edition of DSM "the essential feature of Attention-Deficit/Hyperactivity Disorder is a persistent pattern of inattention and/or hyperactivity-impulsivity that is more frequent and severe than is typically observed in individuals at a comparable level of development" [15]. Currently, to receive the diagnosis, children must, before the age of 7, exhibit at least 6 core symptoms and these symptoms must cause some impairment in at least 2 settings (such as home and school), although severe impairment in one setting can suffice [16].

DSM IV lists 18 core symptoms of ADHD, which are divided into 2 major behavioral domains: (1) inattention and (2) impulsivity-hyperactivity. Among the 9 symptoms of inattention are: often makes careless mistakes, often has difficulty sustaining attention in play or other activities, and often does not seem to listen when spoken to directly. Among the 9 symptoms of hyperactivity-impulsivity are: often fidgets or squirms, often can not stay seated, blurts out, and is impatient.

To bring conceptual order to this heterogeneous set of behaviors, clinicians in the US currently distinguish among 3 subtypes of ADHD: Predominantly Inattentive Type, Predominantly Hyperactive-Impulsive Type, and Combined Type. ADHD Not Otherwise Specified (ADHD NOS) is a fourth category, and includes children who exhibit fewer symptoms of inattention or hyperactivity-impulsivity than children who meet the criteria for one of the other 3 subtypes, but are nevertheless significantly impaired as a result of their symptoms. (More recently, the usefulness of the conceptual order brought by the subtypes model has been questioned [17].)

Findings from genetics and neurobiology have shed some light on the genetic and neurological correlates of the behaviors associated with ADHD [18,19]. For example, as psychiatrist and workshop participant Laurence Greenhill pointed out, researchers recently reported that a gene variant, which codes for a dopamine receptor (the DRD4 7-repeat allele) and was formerly thought to be a genetic marker for ADHD, is also associated with thinning of the cortex in regions associated with attention control [20]. These same researchers also found that among children with ADHD, those with the DRD4 7-repeat allele eventually became more similar to normal subjects than did ADHD children with a different genetic variant. (Brain scanning research has also suggested that the brains of some children with ADHD eventually "catch-up" with the brains of unaffected children [21].) Findings such as the one involving DRD4 may one day help clinicians to identify a class of children with ADHD who are likely, over time, to outgrow their dysfunctional behaviors. But that day has not yet come [19]. We simply do not yet have a genetic test or a brain scan to diagnose ADHD, much less its subtypes.

For one thing, geneticists today are grappling with the fact that, in general, single gene variants by themselves are less helpful in explaining the emergence of complex phenotypes than was once hoped [22,23]. Moreover, neurobiologists are grappling with the fact that variations in single neural circuits are less helpful by themselves in explaining the emergence of complex phenotypes than was once hoped [23]. Indeed, there is increasing agreement that, to understand the etiology of phenotypes as complex as ADHD, it will be necessary to investigate myriad genes, multiple neural circuits, and myriad environmental variables, all interacting over time [24]. This phenomenological and etiological heterogeneity begins to explain some of the disagreement within – and beyond – psychiatry about where the threshold lies between children who should and should not receive the diagnosis.

## The fact that symptoms are dimensional creates a zone of ambiguity and helps to explain disagreements about diagnosis

The creators of the DSM system of diagnosis had several aims. They wanted to develop an algorithm that could quickly and reliably identify individuals who needed help. Such a neatly laid-out system would facilitate getting reimbursement to deliver services. And the careful description of symptoms was intended to help physicians and researchers in different places feel confident that they were indeed studying the same disease entity [15].

While the DSM system achieves those aims, it also entails significant difficulties. First, a single diagnostic label – ADHD – is used to name children who have different collections and levels of symptoms and who suffer different levels of overall impairment. Moreover, as workshop participant and child psychiatrist Gabrielle Carlson suggested, like many conditions, ADHD is expressed differently in different children and it differs in severity from mild, to moderate, to severe (some children with severe ADHD require hospitalization).

Because ADHD does not have a single, simply identifiable form, diagnosing it requires an observer's interpretation. While many physicians will agree that one particular child warrants an ADHD diagnosis and another child does not, many children will occupy what we will call a "zone of ambiguity." Physicians, teachers, and parents may well disagree about whether children in the zone of ambiguity exhibit the symptoms and suffer severe-enough impairment from those symptoms to warrant the ADHD diagnosis. Increases in the rates of ADHD diagnoses and the use of stimulant treatment have fueled the concern that too many children in the zone of ambiguity are today given an ADHD diagnosis rather than considered simply "different" or "spirited," and that drugs are too often the treatment of choice for these children.

The Introduction to DSM IV addresses some of this difficulty by acknowledging that many psychiatric diagnoses give labels to phenomena that are *dimensional*, not *categorical*. Children with and without ADHD do not occupy cleanly separate categories; rather, they occupy different places on one or more dimensions (or continua or spectra) of behavior. Bright lines do not separate children whose attention, impulsivity, or activity levels are normal from those whose are not (as with many disorders, including hypertension and hypercholesterolemia). All children lie somewhere on these behavior spectra and many will fall in the zone of ambiguity. Despite the reminder in its Introduction that psychiatric disturbances are dimensional, DSM is a categorical system. Because DSM is so important for diagnosis and reimbursement, users often adopt its language and categories without recalling its limitations.

According to several workshop members, yet another problem is that, even though DSM explicitly states that diagnoses should only be made if symptoms cause "clinically significant impairment in social, academic, or occupational functioning," clinicians sometimes base their diagnoses on the presence of symptoms alone. In one study, researchers reduced a 16.8% ADHD prevalence rate to 6.8% by adding an impairment criterion [25]. While impairment may be inferred from the fact that parents made an appointment with a health professional, impairment is not always carefully established by the physician making the diagnosis. Reimbursement systems, which usually require a DSM diagnosis, can also encourage clinicians to record a diagnosis of ADHD, even when the severity criteria are not fully met, in order to justify the provision of services. A further problem of a symptom-based diagnosis is that children with many symptoms but less impairment may receive treatment *and *children with fewer symptoms but greater impairment may go undiagnosed and untreated.

Our description of these complexities and the blurriness of the lines is not to suggest that ADHD is not real. The symptoms of ADHD can cause significant suffering in children, families, and schools [26-28] and significant costs to the health care system, education system, juvenile justice system, and employers through parental work loss [29]. We also do not mean to suggest that the DSM descriptions and established diagnostic systems are hopelessly imprecise. Indeed, they are clear enough that raters who are trained to use the same diagnostic system can reach similar conclusions about prevalence rates. Rather, we describe these complexities and the blurriness of the lines to urge us to remember that ADHD is not a unitary, simple thing. Like many other behavioral and emotional disturbances, ADHD is a label for heterogeneous collections of dimensional behaviors that appear to have heterogeneous causes.

To invoke a term that no fewer than three psychiatrists – Michael First, Steven Hyman, and Benedetto Vitiello – used at our first workshop, we need to avoid the "reification" of the DSM categories. These categories are abstractions we have created, not entities we have discovered in nature. Diagnostic categories can be useful tools to help us talk about childhood behavioral and emotional disturbances, but we need to remember that they are tools created by us. We – doctors, parents, teachers, and others – set the threshold between behaviors and moods in need of pharmacological or behavioral treatment and differences that should be left alone or dealt with in other ways.

One explanation for increased rates of diagnosis and stimulant use, therefore, is that we are setting ever lower diagnostic thresholds. This explanation concerned many workshop participants, including sociologist Peter Conrad and pediatrician William Carey. When they see data documenting an increase in diagnostic and treatment rates, they see a troubling decrease in societal tolerance of the behaviors and impairment associated with the ADHD diagnosis.

## Other variables also help to explain why ADHD is diagnosed at different rates in different places

We have already observed that stimulant medications are used at different rates in different countries. Specifically, they are used at higher rates in the US than in culturally similar places like Germany, the Netherlands, and the UK. (Although not perfect, there is a strong correlation between stimulant use and the ADHD diagnosis.) But even within the US there is significant variation in diagnostic and treatment rates. Beyond the phenomenological and etiological complexity and the zone of ambiguity we described earlier, why would rates of ADHD diagnosis be higher in some places than others?

### Variations in diagnostic systems

At least when it comes to understanding the difference between the rates at which ADHD is diagnosed in the US and Europe, it helps to notice that clinicians in those two geographical regions use closely related – but importantly different – systems of disease classification. Whereas clinicians in the US currently use the DSM IV, clinicians in Europe use the 10^th ^edition of the World Health Organization's International Classification of Diseases (ICD 10).

ICD 10 refers to Hyperkinetic Disorder (HD), whereas DSM IV speaks of ADHD. And while DSM IV and ICD 10 use very similar lists of symptoms for ADHD and HD respectively, their approaches to diagnosis are different in some important ways. DSM IV requires a child to exhibit only 6 symptoms in 1 of 2 broad domains (inattention or hyperactivity-impulsivity), while ICD 10 requires a child to exhibit 10 symptoms, including at least 1 in each of 3 domains (inattention, hyperactivity, and impulsivity). Whereas DSM IV requires that some impairment be present in more than 1 setting (school, home, etc.), ICD 10 requires that all criteria must be met in at least 2 settings. In short, the DSM system casts a wider net than does the ICD, so that "ADHD prevalence rates based on DSM-IV are expected to be higher than those based on ICD-10" [30]. Indeed, as workshop participant and child psychiatrist Jörg Fegert noted, the DSM approach produces 3 or 4 times as many diagnoses as does the ICD approach [31].

The ICD and DSM approaches to coexisting conditions in a single child are also importantly different. Under DSM, a child can be diagnosed with ADHD *and *one or more coexisting conditions, such as an anxiety, mood, or developmental disorder. According to ICD, however, if one of those coexisting conditions is diagnosed, then HD cannot be diagnosed.

However, even in the US, where all clinicians presumably use the DSM approach, there is variation. As with other disorders, community-based ADHD prevalence rates from treatment data vary according to demographic factors such as age, gender, and race/ethnicity [32]. As workshop participant and pharmacological epidemiologist Julie Zito added, we have long noticed variation in rates of diagnosis and treatment by age and sex, with higher rates reported in children aged 10–14 compared with children aged 5–9 years, and higher rates in boys.

As with other disorders, there is also considerable regional variation. Workshop participant and child psychiatrist Regina Bussing pointed to Centers for Disease Control data showing that the heaviest use of stimulants to treat ADHD occurs in southern US states, followed by states in the upper Midwest [33,34]. There is also variation within states, and even within counties [35]. Geographic variation in treatment patterns is not uncommon in medicine [36], and is ascribed to a number of factors, including variation in levels of access, rates of occurrence, rates of service utilization, treatment preferences, and clinical practices [37,38].

Laurence Greenhill pointed to still other variables to help explain international and regional variation in diagnosis and use of prescription medication. Clinicians in different places rely on different informants (e.g., parents alone, or parents and teachers) and use different diagnostic guidelines [39] and diagnostic tools (e.g., the ADHD Rating Scale-IV vs. the SNAP IV). Some geographic areas have virtually no child psychiatrists, which means that primary care physicians make almost all the ADHD diagnoses. Physician specialty can affect diagnostic rates because primary care physicians are thought to be at risk for under- and over-diagnosing ADHD [14,40].

### Variations in home, school, and community environments

We also know that children's home, school, and community environments can differ greatly, including their sleep patterns, diets, physical exercise opportunities, and levels of exposure to television and other media. Anthropologist Sara Harkness cited studies she and child psychologist Charles Super have conducted comparing Dutch and American parenting styles: "The Dutch parents we studied were very closely attuned to their children's state of arousal and self-regulation, making sure that the child got plenty of sleep and that the environment was not overly stimulating. For Dutch parents, this was just a normal aspect of good parenting, whereas for American parents this approach might seem somewhat extreme, called for only when the child is really out of control."

Harkness went on to note that Dutch schools are very concerned with each child's ability to pay attention: "Virtually every classroom I visited had two or three children's desks that were placed away from the others (touching the teacher's desk in a couple of cases), in order to help children who seemed more distractible than others." Harkness and Super also noted differences at a systems level; children who had difficulty learning due to behavioral problems were transferred to special schools, and the school day included a lunch break long enough for children to go home and spend time outside in unstructured play. While the environmental causes of the behaviors considered symptoms of ADHD are not well understood, many workshop participants agreed that the child's environment can influence the development of such behaviors.

In addition, because different cultures have what Roy Martin called "different local normative expectations," different environments will be more or less tolerant of active, distractible children, and will be more or less prone to see impairment from those behaviors. To put the point in diagnostic terms: observing the same behaviors at the same rates in children around the world is one thing, but these children will not meet the diagnostic criteria unless they are also impaired by those behaviors in that culture. People in some cultures are also more likely than people in others to seek medical assistance and accept medical (particularly pharmaceutical) treatments [41].

Many workshop participants were concerned that cultural expectations in the US have grown intolerant of children exhibiting the behaviors currently associated with ADHD. Peter Conrad spoke of the "medicalization of underperformance," and psychiatrist John Sadler worried that changes in expectations about the conduct of classroom education and the pace of educational achievement make it more likely that the active, distractible child will be considered a problem. That said, many workshop participants agreed that children with the most severe forms of ADHD would be impaired in virtually any culture, community, or context.

Bearing in mind the myriad factors that can affect how different people interpret the same behaviors, and remembering the phenomenological heterogeneity and etiological complexity associated with ADHD as well as the zone of ambiguity, it is hardly surprising that rates of diagnosis are different in different places. More specifically, setting aside the debates about the particulars of the DSM approach, we can see why there are concerns about both over- and under-diagnosis.

### Over- and Under-Diagnosis

The Great Smoky Mountain study examined the prevalence of serious emotional and behavioral disturbances, including ADHD, in children in the western region of North Carolina [42]. In the study, trained interviewers applied DSM criteria, including the requirement for impaired functioning, to a representative sample of 1,422 children. From these data, the researchers estimated that about 6.2% of children in the community met the criteria for ADHD (a greater number exhibited one or more ADHD symptoms but fell short of the diagnosis). The study then looked at rates of stimulant use and found that 7.3% of children in the study had received stimulants at some time during the 4 year study period. At first glance it might therefore appear that slightly more children received stimulants than met the DSM criteria for ADHD; in fact, over 57% of those who received medication did not meet the criteria.

Two factors explain the Great Smoky Mountain study's findings. First, not all of the children who warranted an ADHD diagnosis had received stimulants; 72.2% of the children who warranted an ADHD diagnosis received stimulants and only 22.8% of children who warranted an ADHD-NOS diagnosis received stimulants. Second, 4.5% of children who did *not *warrant an ADHD diagnosis nevertheless received stimulants. While 4.5% is a small percentage, it is 4.5% of all the children in the study who did not have ADHD, which is a large number. In terms of *absolute numbers*, the study found that more children without ADHD received stimulants than did children with ADHD. The study concluded that in the community (as compared to a rigorous research trial), a significant proportion of children with ADHD do not receive stimulants *and *a significant number of children without ADHD are prescribed stimulants.

It is widely recognized that ADHD is over-diagnosed in some affluent communities, where "local expectations" are such that stimulants are just one more tool to promote performance in "the Academic Olympics" [43]. Because we, the authors of this document, assumed that children living in poverty might be more likely to be judged unruly and therefore be prescribed drugs like Ritalin, we came to the workshop expecting to learn that ADHD is also over-diagnosed in poorer children. We discovered that the issue is a bit more complex. It is true that, in the US, access to mental health services generally decreases with lower economic status. Even though many poor children qualify for publicly-funded programs, such as Medicaid, and therefore for care that compares well with the care offered to economically advantaged children, poor families often under-utilize the services to which they are entitled [34,44-46]. (The exception may be children in foster care, who are almost all eligible for Medicaid, but whose utilization rates are higher than other Medicaid-enrolled children [47]). Add to this complexity that children in poor or wealthy families may well be subject to different "local normative expectations," and we can see how rates of diagnosis might vary by economic status.

## Different views about the proper goals of psychiatry and parenting lead to less or more concern about treating children in the zone of ambiguity

While there frequently will be agreement among experts about whether to diagnose ADHD in children with very *mild *and very *severe *impairment due to the behaviors associated with ADHD, there always will be some children whose symptoms and impairment place them in the zone of ambiguity.

At least in part, views about where to set the threshold for diagnosing ADHD will be a function of peoples' differing conceptions of the proper goals of medicine in general or psychiatry in particular. Some observers are not alarmed by the tendency of medical institutions to treat ever more problems that seem to have more to do with someone's failure to meet social, cultural, or educational expectations than with a failure of physiological function. Others, like sociologist and workshop participant Peter Conrad, who are often alarmed by this tendency, label it medicalization and see both diagnosis and treatment as "social control for deviant behavior" [48].

Sharing the medicalization concern, workshop participants like pediatrician William Carey emphasized that we need to get better at accepting that children come into the world with different temperaments (or behavioral styles) [49]. According to Carey, if we better understand that "normal" includes a wide variety of temperaments, we will also understand that temperamental differences do not necessarily entail either impairment or harmful dysfunction. We then will be quicker to accommodate such differences and slower to treat them with a branch of medicine. Carey is not simply urging caution about using *drugs *to alter a child's temperament; he is urging caution about using *any *means to shape what he urges us to view as normal temperamental differences. He is equally concerned about the overuse of behavioral interventions, which he thinks should be reserved for responding to problematic *behaviors *(e.g. ignoring teacher requests) rather than problematic behavioral *styles *(e.g. a restless temperament) [50].

The authors of this report, and many members of the workshop, share Carey's commitment to tolerating and even affirming a diversity of temperaments. And we recognize that this commitment is, to some extent, rooted in an intuition about our appropriate attitude toward ourselves and the world. It is an intuition about the value of accepting and affirming children "as they are" and allowing them to unfold in their own way, as opposed to seeking to transform them into our vision of how they ought to be [51]. When we speak of Carey's "commitment," and suggest that it grows out of an intuition about what is valuable, we are not suggesting that it is unimportant. We mean only to recognize that, as important as it is, the concern about medicalization does not grow out of reason alone and will not be shared universally. We would, however, emphasize that people who are not alarmed by medicalization *also *proceed from intuitions about what is valuable.

Indeed, other members of our workshop, such as psychiatrist and neurobiologist Steven Hyman, introduced a different intuition about how to respond to children in the zone of ambiguity. He suggested that, whatever the historical goals of medicine, if we can use it to reduce children's suffering and enhance their agency, perhaps we should. He, too, is aware of the importance of letting children be "as they are," but he emphasizes that parents are also obliged to shape their children and improve their chances of living a good life in the culture in which we live. If a choice has to be made between promoting a child's flourishing in our world and accommodating and affirming her temperamental differences, Hyman and many others might choose the former.

Neither the authors of this document nor anyone else has the "view from nowhere" that would be required to pronounce which of those positions is right. We seek only to emphasize that those who would set the threshold for the diagnosis of ADHD low and those who would set it high both appeal to intuitions and values. Neither side appeals to facts alone.

## There is nothing "mere" about social constructions

Because the ADHD diagnosis involves interpretations and values, and because the rates of ADHD diagnosis vary from place to place, it has been argued that ADHD is not a real disorder, but is instead a cultural or social construct [7]. A recent meta-analysis that examined studies of prevalence rates in different countries, and the published commentaries that accompanied it, grappled with just that charge.

In 2007, Guilherme Polanczyk et al. published a widely cited article that analyzed much of the extant literature on the prevalence of ADHD/HD [30]. They found studies reporting prevalence rates ranging from a low of 1% to a high of 20%. But in their analysis they argued that, if, in addition to taking into account the geographic location of the study, one takes into account the methodological differences among the investigators – the different diagnostic criteria that the studies used, who reported on the symptoms, and how much impairment was required for diagnosis – the worldwide prevalence of ADHD/HD is 5.3%.

In a commentary accompanying the Polanczyk study, Terrie Moffitt and Maria Melchior wrote that the study shows that ADHD is "a bona fide mental disorder (as opposed to a social construction)" [52]. We would offer a different interpretation, one that Olavo Amaral in fact offered in a letter responding to the Moffitt-Melchior commentary. Amaral wrote: "The concept of a disorder and its diagnostic criteria are social constructions by definition, and the fact that a group of symptoms has a constant geographic prevalence has little to do with what leads these symptoms to be considered a diagnostic entity" [7]. As he points out, twin pregnancies, for example, are largely equally prevalent across the world, but whereas having twins can still be a source of shame in some South American countries, it tends to be a source of pride in North America. The same phenomenon is "constructed" differently in different places. Workshop participant and child psychiatrist Benedetto Vitiello put the same point in subtler terms: even where culture does not affect the frequency and presentation of a certain behavior, it certainly influences the local interpretation of that behavior. It may be that a group of raters trained to apply DSM criteria would diagnose children with ADHD at about the same rate in different countries. But such a finding would not tell us that these children were "really" disordered if some of them are nevertheless considered normal enough (i.e., not disordered) in their own countries. Determining which children are "really" disordered will always be in part a function of the culture in which the child lives.

Polanczyk et al.'s response to Amaral's letter is worth noting [53]. In emphasizing the similarity in the prevalence of ADHD across cultures, they said that they intended to help reduce the stigma associated with the ADHD label. They assumed that in establishing the fairly uniform prevalence of ADHD behaviors across cultures they were demonstrating the reality of the disorder. In defeating the claim that ADHD is "merely" a social construction, they aspired to get treatment to children who need it – especially, they emphasized, poor children. Second, they argued that, even though different cultures may interpret certain universal phenomena differently, some cultures are more correct in their constructions. To make this point, they suggest the example of obesity: Yes, it may be constructed "positively" in some Pacific Island cultures and "negatively" in North America, but "the link between obesity and several adverse outcomes is well established, supporting its validity as a medical condition."

While participants in our workshop would argue that we do not understand the causes and effects of the behaviors associated with ADHD as well as we understand the causes and effects of obesity, many accepted that the behaviors associated with ADHD appear in children across the globe, whether at the same or slightly different rates. And all believe that we have a moral obligation to help children who suffer from harmful dysfunction as a result of these behaviors – especially those who currently are underserved. But where to set the threshold between normal variation and ADHD (or obesity) that deserves treatment is up to human beings, whose perspectives and values will differ. The threshold is *not *inscribed in nature, as is made clear by the fact that two reasonable and widely used diagnostic systems, DSM IV and ICD 10, draw it at different places.

Recognizing that it is up to human beings operating in particular cultural contexts to decide where to set the threshold between disordered and normal behavior does not commit us to viewing ADHD as "merely" a social construction. ADHD *is *a social construction; what it is depends on our interpretations of natural phenomena. But there is nothing "mere" about it. Social constructions are as real as any other feature of our lives.

Once we accept that there is no clear line between children with and without ADHD, and that this line must be articulated by physicians, parents, teachers, and others in society, we still need to ask, What should we do to help children who we deem to be impaired by their ADHD behaviors?

## The facts surrounding the most effective treatment of ADHD are complicated and incomplete

There are many possible responses to the behaviors associated with ADHD, from changing the child's sleeping and eating patterns, to classroom interventions, to medication. Only some of these responses require the help of medical professionals. Here we refer to all medical responses as "treatments." The two main treatments offered by health professionals to children diagnosed with ADHD are medications and behavioral therapy. The stimulant Ritalin (methylphenidate) was approved by the FDA to treat the symptoms of ADHD in children in 1955 and behavioral treatments have been developed and studied over the past several decades [54]. Of the two treatments, stimulants are administered most frequently [39].

In 1992, the National Institute of Mental Health and the Department of Education cosponsored a randomized clinical trial to compare the long-term efficacy of these two treatments. Over the course of 14 months, researchers observed children with ADHD who were being treated with either: (i) the researchers' carefully crafted regimen of *medication*; (ii) intensive *behavioral *treatment (with responsibilities for the child, parents, teachers and teacher-aids, and therapists); (iii) *combined *medication and behavioral treatment; or (iv) standard community care (i.e., whatever providers in that child's community offered to children with ADHD).

The initial, highly influential conclusions of this Multimodal Treatment Study of Children with ADHD (MTA) were published in 1999. After 14 months, MTA investigators concluded that, while all 4 treatment options showed sizable reductions in symptoms, their finely tuned regimen of medication alone was superior to the other 3 arms of the study for treatment of ADHD symptoms [55]. The MTA group wrote: "If one provides carefully monitored medication treatment similar to that used in this study as the first line of treatment, our results suggest that many treated children may not require intensive behavioral interventions" [55].

Following publication of the initial MTA findings, enthusiasm for drug treatment appeared in high-profile practice guidelines (including those from the American Academy of Pediatrics [56] and the American Academy of Child and Adolescent Psychiatry [57]). The consensus seemed to have become that drugs alone are an effective treatment for ADHD, with behavioral approaches a possible adjunct.

However, according to some of our workshop participants, including psychologists William Pelham and George DuPaul, the drugs-first approach is mistaken. They point out that when MTA followed-up with their participants, 22 months after the study had ended, combined and behavioral treatments were as effective as medication alone at reducing ADHD symptoms. Perhaps more importantly, they (and William Carey) argue that reduction of core ADHD symptoms alone is not sufficient to determine effectiveness [58].

While it was clear to the MTA researchers that at 14 months those children taking their carefully managed stimulant regimen exhibited the greatest reduction in ADHD symptoms, they also recognized at that time the benefits of combination (drug and behavioral) therapy. In a press release accompanying the initial findings, NIMH wrote: "for some outcomes that are important in the daily functioning of these children (e.g., academic performance, family relations), the combination of behavior therapy and medication was necessary to produce improvements, and families and teachers reported somewhat higher levels of consumer satisfaction for those treatments that included behavioral therapy components. Furthermore, the combination program allowed children to be treated over the course of the study with somewhat lower doses of medication" [59]. The study also found that for children with coexisting conditions, combined treatment was superior at controlling ADHD symptoms. It seems to us, the authors, that these findings were not given sufficient attention when the initial MTA findings were published.

Even if one focuses only on improvement in ADHD symptoms, later reports from the MTA study throw some doubt on the superiority of medication-only treatment. When MTA researchers followed up with the children nearly 2 years after the study ended, they found that those who had originally been assigned to the medication arm of the study no longer outshone those in the other three arms. In fact, all three groups showed similar levels of ADHD symptoms. "By 36 months, none of the randomly assigned treatment groups differed significantly on any of the five clinical and functional outcomes" [60].

Pelham and DuPaul also argue from their own data and experience that *combined *medication and behavioral interventions may produce significantly more improvement in key domains of daily life functioning than medication alone, and that behavioral treatment can make it possible to use lower "doses" (or intensity) of the drugs [61,62]. Lower doses of medication have fewer side effects and a better safety profile. In response to concerns about the financial and time commitment for behavioral treatment, they point out that some families report significant improvements with less intensive behavioral treatment than was used in the MTA study, and that behavioral treatment can be tapered off; after an initial intensive period, children, parents, and teachers learn new skills and behavioral treatment is incorporated into their lives and work, whereas it is usually assumed that medication will need to be taken long-term.

In addition to behavioral treatment's success at improving ADHD symptoms, Pelham and DuPaul cited data showing that parents and children generally prefer treatment regimens that include, or are focused on, behavioral interventions [63]. There is also evidence that behavioral interventions are more likely than pharmacological ones to lead to permanent improvements in aspects of a child's overall functioning (more on this below). In fact, Pelham and DuPaul are so impressed by the efficacy of behavioral treatments that they argue for using them as first line treatment for many children with ADHD. On this approach, treatment of children with mild or moderate ADHD would begin with behavioral treatment (at home and school). Physicians would, only as necessary, add low doses of medication.

### The medication approach

To begin to understand the apparent discrepancy between the initial findings of the MTA and current common practice, on the one hand, and the latter findings of the MTA and Pelham and DuPaul on the other, it helps to recall how stimulant medications work. Stimulant drugs, like many medications used in pediatric psychiatry, can reduce the severity of, or even eliminate, symptoms. But they do not "repair" or "treat" the underlying causes in the brain of those symptoms. Stimulants can reduce a child's inattentiveness and hyperactivity, but can not by themselves teach the child to control his or her attention or activity levels. Further, relief of symptoms does not necessarily mean improvement in the overall functioning of the child. It is of paramount importance to recognize that how one defines efficacy – whether one measures only reduction in symptoms or also improved academic achievement, improved peer and family relations, improved classroom behavior, etc. – can determine which treatments one considers effective. Treatments that improve symptoms alone do not satisfy those who, like Pelham, believe that "minimization of impairment in daily life functioning and maximization of adaptive skills" ought to be the goal.

Some workshop participants suggested that by reducing symptoms, stimulants should make it easier for children to "learn how to restrain their impulses" or "get ready to learn behaviorally." However, as an empirical matter, as soon as the drug treatment stops, many children return to the behaviors of their original, un-medicated state. As Steven Hyman observed, "cognitive control of behavior doesn't get a boost from these months or years on medication." (This finding did not surprise some workshop participants, who pointed out that a diabetic child who stops taking insulin returns to an uncontrolled diabetic state.)

Importantly, it is not yet established that a reduction in ADHD symptoms necessarily leads to the hoped-for improvements in academic achievement [64]. Medication can "produce acute, short-term improvements in on-task behavior, compliance with teacher requests, classroom disruptiveness, and parent and teacher ratings of ADHD symptoms" [65]. And, as Benedetto Vitiello pointed out, there is evidence that stimulants help to improve school-work accuracy and productivity. But despite these improvements, Vitiello said that researchers do not currently have sufficient data to conclude that improving attention to accuracy or productivity translates into long-term improvements in academic achievement (understood as improvements in standardized test scores or ultimate educational attainment) [64]. Although symptom reduction may be a relief for the teacher, child, and parent, George DuPaul also agreed that it does not usually translate into long-term improvement in academic performance.

No one is quite sure why a reduction in ADHD symptoms does not translate into long-term improved academic achievement. As a partial explanation, Pelham noted that while attention and productivity are necessary for learning, they are not sufficient (attention, productivity, and learning are different processes). Other workshop participants added that a reduction in ADHD symptoms cannot erase any learning disability that a child might have nor make a child more intelligent. One thing, however, is clear: parents, teachers, and physicians all deserve to know the state of the evidence.

In addition to concerns about long-term efficacy, worries about adverse drug effects persist, even though stimulants have been used for more than half a century. According to several reports [66], long-term stimulant use can slow physical growth by 1.2 cm per year [67] and slightly increase blood pressure and heart rate, although the clinical implications of these increases are unclear [68]. Rare instances of sudden deaths have been reported in children receiving stimulants; however, no causal inference has been drawn from these reports because a high proportion of the children also had structural heart abnormalities [69]. Long-term use of stimulants can also increase insomnia or decrease appetite [70]. While most available data do not suggest that therapeutic use of stimulants increases the risk for subsequent drug abuse [71], all current studies have methodological limitations that prevent drawing a definitive conclusion on the link between stimulants and substance abuse. Clearly, more research is needed on the long-term effects of stimulants on the developing brains of the ever-younger children who receive them.

### Behavioral approaches

The potential for adverse drug effects, no matter how small, is one reason why some people invoke the principle of "do no harm" – and urge beginning with behavioral treatments. Proponents also point to multiple studies showing that behavioral treatments are more effective than drugs alone at improving the overall functioning of children with ADHD [72].

Advocates for "behavioral treatments" are referring to a set of interventions that include teaching parents how to better parent a child with an ADHD diagnosis, teaching teachers how to better teach children with ADHD, and helping children take responsibility for monitoring and managing their own behavior. Parents and teachers post rules, adjust workloads, provide choices, reinforce good behavior, and offer special tutoring [73]. The MTA study described above showed that this kind of behavioral treatment significantly reduced the symptoms of ADHD and improved some aspects of the child's overall functioning (with and without low doses of concurrent medication) [55]. Nearly two years after MTA's behavioral treatment finished, there had been no loss in its effectiveness and the majority of children who received it were still unmedicated [60]. Behavioral treatments show an effect even after the formal therapy ends because, in theory and to a surprising extent in practice, parents, teachers, and children continue to implement what they learned. (Like dieting and exercise to combat obesity, behavioral treatments only continue to work if individuals continue to follow the new behaviors.) However, while behavioral treatments are associated with improvements in aspects of overall functioning, such as parent-child interactions and a reduction in oppositional-defiant behavior, their impact on long-term academic achievement has not been carefully studied [64].

While there was enthusiasm among many workshop members for using behavioral interventions as the first line of treatment – and when necessary for combining behavioral and pharmacological approaches to maximize functional improvements – there was also a keen sense of the challenges inherent in behavioral approaches. As child psychiatrist Gabrielle Carlson pointed out, because they require a lot of parents, behavioral interventions can be difficult for some parents to carry out, including those who themselves struggle with ADHD.

In a similar vein, Carlson, Martin, and Super each emphasized that behavioral approaches may impose more demands on already overburdened teachers, suggesting that making behavioral treatments effective will mean addressing education at a systems level, rather than simply at the level of the individual teacher and student. Martin also expressed concern about whether it is realistic to hope to "scale up" the behavioral programs described by researchers like Pelham. Sara Harkness granted that for behavioral treatments to be adopted by parents and teachers – and she is confident that they can be – "a change in mindset is required, and structural changes in school schedules as well as family routines should be brought into the discussion."

During our discussion of the costs of behavioral approaches, Pelham argued that behavioral approaches would actually save money in the long run because the changes they bring about are long-lasting. When combined with medication, behavioral treatments can also allow for lower doses of medication to be used, thereby saving on medication costs. He also argued that, while the behavioral treatment used in MTA was extremely intensive, lower "doses" of behavioral treatment will suffice for many children with mild to moderate ADHD.

Many workshop participants agreed that failing to respond to ADHD – whichever treatments are offered – also carries costs, to the health care system for associated injuries and medical problems, to the education system, and to the juvenile justice system [29]. Acknowledging the costs of ADHD, however, does not tell us what is the most effective, including most cost-effective, means of treating or otherwise responding to children with an ADHD diagnosis.

Finally, pediatrician Kelly Kelleher asked the group to consider, quite aside from cost, how very difficult it is to get from the state-of-the-art treatments administered by pediatric psychiatrists working in research settings to the treatments that average children receive in community practices. As he reminded us, it would be irresponsible to forget the size of the gap between "academic expectations and practice in the trenches."

## The pharmacological and behavioral approaches emphasize different values

Children who are impaired by the symptoms of ADHD deserve access to thoughtful, evidence-based treatment. While there is some agreement about clear-cut cases (where no medical treatment need be offered, relying instead on changes to the home or school environment, or where drug and behavioral treatments should be offered), it is inevitable that physicians will sometimes disagree about *how *to respond. Just as values play an ineliminable role in reaching decisions about where to set the threshold for the diagnosis of ADHD, so too are they implicated in decisions about which treatment would be best for a particular child [74].

As mentioned above, some of the value differences that arose in discussion of the proper *goals *or purposes of child psychiatry in general, also arose in discussion of the specific *means *used to treat many childhood disturbances: psychotropic medications. Pharmacological epidemiologist Julie Zito argued that some children will simply outgrow and learn to control negative behaviors and therefore that we should sometimes "let nature take its course." Other workshop participants, such as pediatrician Lawrence Diller, argued that when choosing between drugs and behavioral interventions, we should begin with those behavioral interventions that have proven efficacy. Zito's and Diller's preferences can be explained by concerns about the side-effects and safety of drugs, as well as their long-term efficacy. But we think that they also may be explained by examining the different values expressed in drug treatments on the one hand, and behavioral treatments on the other.

Whereas medications tend to emphasize the value of efficiency (insofar as they are quicker acting and, in the short-term, cheaper), behavioral interventions tend to emphasize the value of engagement (insofar as they require the child to engage with parents, peers, teachers, or therapists, and they require these others to engage with the child and with his environment) [75]. Because they do not seem to locate the "problem" in the child's body, but instead in the interaction between the child and his home, school, and social environments, behavioral interventions prompt us to notice the importance of the child's environment and take steps to improve it. They also can help the child learn to think of himself as a moral agent, as someone who can learn how to change [75].

Critics of pharmacological means have also raised the concern that stimulants separate or alienate the child from who he really is, thereby undermining or diminishing his authenticity [76]. Workshop participant and psychologist Ilina Singh is investigating the authenticity concern by interviewing children and their parents about the ADHD diagnosis and stimulant medication [77]. However, her preliminary results show that parents and children do not say that medication alienates the child from himself, but rather that by helping the child to overcome his "inner badness," the drugs *empower *him. (Or, as the language of authenticity would have it, drugs allow the child to become "who he really is.") Singh's unfolding work is offering a complex account of how parents and children speak about the relationship between the self, their ADHD diagnosis, and medication.

Like some of the parents and children Singh interviewed, some members of our workshop also tend to be impatient with the concern that drugs will alienate us from ourselves or undermine our agency. They even invoked Gerald Klerman's phrase "pharmacological Calvinism" [78] to suggest that opposition to pharmacological interventions is motivated by an unreflective, quasi-religious commitment to the value of suffering. According to the "pharmacological Calvinism" charge, it does not matter which means we use to reduce problematic behaviors. What matters is the end or goal of the intervention: insofar as drugs and behavioral interventions both aim at changing brain wiring, there is no good reason to automatically prefer one over the other.

It is important to notice that people who are more and less comfortable using drugs to treat ADHD do not so much hold altogether different values as they emphasize different values [51]. Both sides want to use the treatment that is best for the child and her family. But just as determining what is "genuine" impairment (and therefore who warrants the ADHD diagnosis) and what is "merely" a temperamental difference (and therefore does not require diagnosis or treatment) involve values, so too does deciding whether a drug, a behavioral treatment, or some other response is best for a particular child. We see no "crisp" [79] solution to disagreements that emphasize different values. Instead, we must accept a certain amount of variation, uncertainty, and ambiguity when it comes to diagnosing and treating children who exhibit the symptoms associated with ADHD.

## Truly informed decision-making requires grappling with complicated and incomplete facts regarding diagnosis and treatment

For the many reasons we have already discussed (including phenomenological heterogeneity; etiological complexity; differences in diagnostic and reimbursement guidelines and tools; and differing cultural and educational expectations of children), there always will be a zone of ambiguity: a range of cases where it is just not obvious whether we should consider a given child as disordered and in need of treatment or temperamentally different and in need of toleration and adaptation, or something in between. How we conceive of that child is important, insofar as labeling her as disordered will entail responding to her first with treatment, and labeling her as different will entail responding to her first by placing her into, or creating for her, a more welcoming environment. We emphasize "first" because these two responses are not mutually exclusive. In those cases where it is decided that treatment is appropriate, there may be reasonable differences of opinion about whether to begin with drugs, behavioral interventions, or a combination.

Gabrielle Carlson suggested that the severity of a child's impairment can guide treatment choices. Children with *very mild *impairment might best be viewed as needing tolerance of, and adaptation to, their temperamental differences rather than drugs or intensive behavioral treatment. Children with *mild *ADHD might benefit from classroom adaptations, improved structure and consistency at home, and some teaching of organizational skills, but should only be offered a stimulant if the behavioral interventions fail. Children with *moderate *ADHD should receive behavioral treatment and might warrant drug treatment during school hours. Children with *severe *ADHD should be offered intensive behavioral treatment and a drug treatment. Carlson's suggestion emphasizes that decisions about whether to treat and how will vary depending on assessments of the severity of the child's symptoms and impairment. Her suggestion, of course, does not obviate the need for these thresholds and categories.

But how should we make decisions in individual cases? In short, parents – and, the child to the extent that she is able – with input from medical, behavioral, and educational professionals, should consider the relevant complexities and make decisions that are as truly informed as possible. As psychiatrist Jefferson Prince pointed out, families have to recognize that, because our understanding of these behaviors and what works to treat them is evolving, many families will to some extent always be engaged in an experiment with their doctors to understand their child's impairment and to determine how best to respond to it. Though it may be difficult or unsettling for families and even physicians to face such ambiguity and uncertainty, the principle of respect for persons requires physicians to help families do exactly that.

Given that many children can understand and form views about their diagnosis and treatment, should they have a say about which treatment they receive? The answer likely depends on the age and maturity of the individual child. According to workshop participant Jörg Fegert, whereas school age children say they want to be informed by their parents about decisions that directly affect them, adolescents say they want to be actively involved in the decision making process. Getting *assent *from school-age children and *informed consent *from adolescents under some circumstances squares well with the 2001 recommendations by the American Academy of Pediatrics [80] and with work done by other researchers from childhood studies, such as Priscilla Alderson [81]. Nonetheless, we recognize that the questions regarding the role of children in decisions about their own treatment – especially for emotional and behavioral disturbances where the child's very capacity to understand their disorder may be at issue – require much more research and reflection, and will likely vary according to the maturity, insight, and abilities of the individual child. At least one workshop member, Gabrielle Carlson, lamented that, whereas we do know something about how parents feel about treating children for ADHD [82], we know little about what children feel about their own treatment. Fortunately, that situation is changing [77,83].

Letting families decide hard cases is not a magic bullet. Parents, whether they are together or separated, can disagree about which response is best. Our workshop participants did not discuss at length what should happen when parents, parents and children, or families and physicians reach different conclusions about diagnosis and treatment. What little discussion we had on this topic was inconclusive. For example, one physician said that he would treat a child only if both parents agreed, while another physician said he would treat even if only one parent requested it.

All agreed, however, that making decisions that are as truly informed as possible requires good information about the child's symptoms and impairment as well as about the impact of treatments, which can take time. To save time, Kelly Kelleher suggested gathering detailed information from multiple sources about a child's symptoms and impairment and about the day-to-day efficacy of treatments through cell phones, web-interfaces, and other technologies. With technology, a team of mental health providers can gather more detailed information than they would access through one-on-one interviews, make better diagnoses, and more carefully monitor children's reaction to treatment. Technology can even remind children or parents to take medications [84]. Kelleher also urged us to abandon our nostalgia for solo practitioners and appreciate the benefits of having patient information shared across large, integrated, technologically savvy systems of care. He argued that the privacy of sensitive medical information can be protected while nevertheless enabling providers to nimbly and quickly communicate with children, their families and teachers, and to follow a child's progress.

Clearly, making decisions that are as informed as possible also requires access to information that is as objective as possible. While workshop members are keenly aware of the great good that pharmaceutical companies do, they are also profoundly concerned about the threat that industry funding of research and relationships between industry and researchers pose to the creation and dissemination of scientific information [85]. The risk that drug companies might suppress or delay release of data from trials that reflect badly on their product or subtly influence the judgment of those charged with designing or interpreting data was deeply concerning to some workshop participants, as was aggressive marketing to health professionals and consumers of pharmaceuticals and the conditions they treat. Any entity or practice that diminishes the quality of the information available to professionals and patients also diminishes the ability of families to reach decisions that are as informed as possible.

Finally, workshop participants returned to a basic issue that, like others discussed above, is not unique to ADHD. Many workshop participants believe that there is a system-wide push towards the diagnosis of mental disorders and the use of drugs in preference to behavioral therapy because drugs are easier to administer and, perhaps wrongly, considered cheaper. Much like other chronic illnesses in the US, ADHD is often addressed in primary care settings where practitioners are under pressure to see many patients quickly and face restrictions on what third-party payers will reimburse. Not only is a diagnosis needed in order to access treatments, but payers often place limits on the number of therapy sessions that will be reimbursed, but not on the number of prescriptions. As workshop participant John Sadler put it: "The economics are simple: we prescribe more drugs in the US because [third-party payers believe] they are cheaper than costly behavioral interventions."

Given the prevalence of ADHD, philosopher and workshop participant Bonnie Steinbock also wondered whether a public health approach, which sought to understand the underlying causes and focused on prevention, might be warranted. Steinbock argued that an analogy might be made with obesity, rates of which have soared in the past few decades. If we understood better the causes of ADHD, we would not simply focus on diagnosis and treatment, but also on policy changes. That is, she brought us back to what Harkness and Super call the child's developmental niche [86], asking us to focus on what can be done at a systems level to improve children's environments so that they are either less likely to develop the behaviors associated with ADHD or are more likely to flourish despite those behaviors.

## Concluding observations

1. The ADHD label refers to a heterogeneous set of symptoms that cause impairment.

a. Until we have valid biological markers, diagnosis will depend upon observers' interpretations of children's behavior.

2. The fact that symptoms are dimensional (rather than categorical) helps explain disagreements about diagnosis.

a. Most children will express some of the behaviors associated with ADHD, but not all children are impaired as a result.

b. While it will sometimes be clear to virtually all observers that one particular child is impaired and another child is not, the dimensional nature of behaviors means that there will be a large *zone of ambiguity*: i.e., a significant number of cases in which reasonable people will interpret the same child's behavior differently.

3. ADHD is diagnosed at different rates in different places.

a. Variation in rates of diagnosis is due to many variables, from different professional systems of diagnosis (e.g., DSM vs. ICD) to different styles of parenting and expectations of children.

b. Using DSM IV criteria, ADHD is both under- *and *over-diagnosed.

4. People hold different – and reasonable – views about the proper goals of psychiatry and parenting, and thus worry less or more about treating children in the zone of ambiguity.

5. There is nothing "mere" about social constructions.

a. To recognize that there is no bright line written in nature between impaired and unimpaired children – to recognize that it is up to human beings to choose who should receive a diagnosis and who should not – is to acknowledge that ADHD is "a social construction." But acknowledging that does not make us diagnostic nihilists; rather, it means we understand that because nature does not show us where that line is, it is our weighty responsibility to decide where to draw it.

6. The facts regarding different treatments are complicated and incomplete.

a. When a diagnosis is made, the facts surrounding the most effective treatment strategy are, unfortunately, less certain than caring physicians and distressed families would wish. For one thing, "efficacy" can be defined in different ways, from reduction in symptoms to improvement in academic achievement and peer and family relations. Recent research suggests that while stimulant medication is often effective in the short term at reducing the core symptoms of ADHD it is not always effective in the long term at improving a child's overall functioning. Moreover, it has been shown that behavioral treatments, alone or in combination with low doses of medication, can be effective in the long term at reducing the core symptoms of ADHD *and *at improving many aspects of overall functioning.

7. People hold different – and reasonable – views about the proper means for psychiatry to employ in the treatment of ADHD.

a. People who favor either medication or behavioral treatment can disagree about both the facts – which treatment most effectively reduces symptoms and improves overall functioning – and values – which treatment most effectively respects individual temperaments and improves children's lives.

8. Truly informed decision-making

a. Parents (and to the extent they are able, children) should be provided with the facts they need to make truly informed treatment decisions. Because many children will fall within the diagnostic zone of ambiguity, and because choosing a treatment plan requires grappling with uncertain facts about efficacy and long-term effects as well as with important values, that process will sometimes be difficult. To be truly informed, that process must be as insulated as possible from financial interests.

b. The current US health care and education systems tend to favor pharmacological treatments, which are believed to be less expensive than behavioral treatments. Scaling up behavioral approaches would indeed require initially costly changes in classrooms, schools, and the education system, as well as in reimbursement policies. It would also entail opportunity costs to parents and children, who would need to work at learning new behavioral approaches. Alone or together with pharmacology, behavioral approaches may nonetheless more effectively improve overall functioning than pharmacological treatments alone.

c. Rather than debating whether medication is good or bad in itself, we should continue the dialogue about how to introduce changes in families, classrooms, schools, health care systems, and cultures so as to reduce the incidence of ADHD behaviors and reduce the likelihood that children who do have those behaviors will be impaired. When families consider it necessary to enlist medical assistance in treating impairing behaviors, they should be carefully informed of the benefits and limitations of medication *and *behavioral therapy.

## Authors' contributions

Both authors contributed equally to all aspects of this article.
